# Local application of statins in the treatment of experimental periodontal disease in rats

**DOI:** 10.1590/1678-77572016-0149

**Published:** 2017

**Authors:** Bianca Fernanda Espósito SANTOS, Eduardo Quintão Manhanini SOUZA, Maísa Ribeiro Pereira Lima BRIGAGÃO, Daniela Coelho de LIMA, Leandro Araújo FERNANDES

**Affiliations:** 1 Universidade Federal de Alfenas, Faculdade de Odontologia, Alfenas, MG, Brasil.; 2 Universidade Federal de Alfenas, Faculdade de Odontologia, Departamento de Clínica e Cirurgia, Alfenas, MG, Brasil.; 3 Universidade Federal de Alfenas, Faculdade de Odontologia, Departamento de Bioquímica, Alfenas, MG, Brasil.

**Keywords:** Periodontitis, Alveolar bone loss, Simvastatin

## Abstract

**Objective:**

The objective of this study was to evaluate the local effects of statins as adjuvants for treatment by scaling and root planing (SRP) of periodontal disease induced in rats.

**Material and Methods:**

Ninety rats were used in the present experiment. Periodontal disease was induced in all animals using a cotton thread placed in the left first mandibular molar. After 7 days of induction, the bandage was removed and the animals were divided into three groups: 1) NT group (n=30), no treatment; 2) SRP group (n=30): SRP and irrigation with control gel; 3) S group (n=30) - SRP and irrigation with Simvastatin. Ten animals from each group were euthanized at 7, 15 and 30 days after treatment. Gingival biopsy specimens were processed to analyze the expression of matrix metalloproteinase 8 (MMP-8). The mandibles were removed and submitted to radiographic and laboratory processing for histometric analysis.

**Results:**

The S group showed a significantly lower expression of MMP-8 compared to NT and SRP groups in all experimental periods. In the radiographic and histometric analyses between the groups, S group showed a significantly lower bone loss (BL) compared to NT and SRP groups in all experimental periods.

**Conclusions:**

Within the limits of this study, it can be concluded that locally applied statin was effective as an adjuvant treatment for SRP in rats with induced periodontal disease.

## Introduction

Periodontal disease (PD) is a chronic infection produced by Gram-negative bacteria with high prevalence levels^2^. It occurs in gingiva in response to bacterial antigens of dental plaque, which accumulate along the gingival margin. Its first manifestation is gingivitis, characterized by redness, swelling, recession and gum bleeding. If not treated early, it can progress to periodontitis^5^.

Type I and III collagens produced by both the periodontal ligament and by gingival fibroblasts are the predominant components of the periodontium extracellular matrix. Initial cleavage of the periodontal ligament and gingival collagen is a key component of the active and progressive periodontal lesions caused by interstitial collagenases, derived from host cells^8^.

Enzymes called matrix metalloproteinases (MMPs) perform the degradation of collagen fibers embedded in the tooth root, which allows both apical migration and lateral extension of the pouch epithelium. The clinical sequelae of this process are the pathological increase in collagen destruction with the insertion loss and formation of periodontal pockets^8^.

According to a study conducted in 1999^26^, the matrix metalloproteinase 8 (MMP-8) is the main interstitial collagenase in the gingival crevicular fluid (GCF) of patients with chronic periodontitis as well as in the peri-implant sulcular fluid of patients with peri-implantitis, representing 90-95% of collagenolytic activity among the collagenases involved in this process.

Treatment of periodontal disease is based on the elimination of pathogenic subgingival microbiota by scaling and root planning (SRP)^15^. However, mechanical therapy alone may be unsuccessful to eliminate pathogenic bacteria for they are usually located within either soft or hard tissues, or even in areas that are inaccessible to periodontal instruments, such as areas of furcation and root depressions^2^. Due to these limitations, supporting methods for conventional periodontal therapy have been studied^7^ ^,^ ^27^. Given the prominent role of the host as a main destruction component of soft and hard tissues seen in periodontitis, therapeutic strategies, such as pharmacological agents, have been highlighted as a new treatment approach^7^ ^,^ ^27^.

Statins are widely used drugs for lowering cholesterol; however, a bone formation induction was observed not only in tissue cultures but also in mice and rats^20^. That fact aroused great interest in the scientific community by considering the possibility that these drugs could be used in bone diseases, such as periodontal disease^20^.

Among the various statins available, there is Simvastatin, which has been widely used in clinical practices to control cholesterol levels. Despite, in addition to its lipid-lowering function, this statin is notable for other side effects, including anti-inflammatory^27^, immunomodulatory, and antioxidant properties, besides the promotion of angiogenesis and increased differentiation of osteoblasts, leading to bone formation^20^ ^,^ ^27^. These properties provide great potential for statins to modify the course of chronic inflammatory diseases^3^, among which periodontal diseases can be included.

Thus, the aim of this study was to evaluate the local effects of statins as an adjuvant treatment to scaling and root planing of periodontal disease induced in rats.

## Material and methods

### Animals

This study was approved by the Ethics Committee on Animal Use (CEUA) of the Federal University of Alfenas-MG - UNIFAL, following the standards adopted by the Brazilian College of Animal Experimentation (COBEA) by number 605/2014. The sample size was determined on a sample population of 117 male rats with an error margin of 5%, heterogeneity of 50% and a 95% confidence level. Considering these parameters, the sample consisted of 90 male rats (*Rattus norvegicus albinus*, Wistar) weighing approximately 200 to 250 g, with 2-3 months of life, from the Central Vivarium of the Federal University of Alfenas-MG - UNIFAL. They were kept under standard conditions with water and food *ad libitum*, at room temperature and with a clear light/dark cycle of 12 hours.

The animals were randomized divided into groups according to a table generated by a computer program.

### Induction of experimental periodontitis

Periodontal disease was experimentally induced by initially performing pre-anesthesia via intramuscular injection of 0.14 mL/kg of ketamine hydrochloride (Rhobifarma Pharmaceutical Industry Ltd.; Hortolândia; SP; Brazil) and 0.06 mL/kg of xylazine hydrochloride (Rhobifarma Pharmaceutical Industry Ltd.; Hortolândia; SP; Brazil) to animals up to 225 g. For animals between 225 and 250 g, the dose used was 0.18 mL/kg of ketamine hydrochloride (Rhobifarma Pharmaceutical Industry Ltd.; Hortolândia; SP; Brazil) and 0.08 mL/kg of xylazine hydrochloride (Rhobifarma Pharmaceutical Industry Ltd.; Hortolândia; SP; Brazil). With the aid of modified forceps, a no. 10 cotton thread (current cotton no. 10; Coats Corrente; São Paulo; SP; Brazil) was adapted around the left first mandibular molars and kept in position by means of surgical knots^13^.

### Study design/Local treatment

After 7 days of induction and progression of PD, the ligature was removed^10^. Then, the animals were randomized into three groups of 30 animals each according to the following local treatments: 1) NT group (n=30), no treatment; 2) SRP group (n=30): SRP and irrigation with control gel; 3) S group (n=30) - SRP and irrigation with Simvastatin.

SRP procedures were performed using 5/6 Mini - Five Gracey curette (Hu-Friedy Mfg. Corporation, LLC; Chicago; USA) with three horizontal movements in the free faces in the mesial-distal direction; and three vertical movements in the interproximal faces in the occlusal-cervical direction^9^.

Natrosol and Natrosol+Simvastatin gel solutions were slowly poured into the periodontal pocket in a single application using a syringe (1 mL) and needle for insulin (13 mm x 0.04 mm) (Becton Dickinson Industry Surgical. Ltd.; Curitiba; PR; Brazil) without bevel. During the treatments, the oropharyngeal region of each animal was protected with a sterile gauze, thereby preventing the intake of any gels and preventing systemic action of Simvastatin.

The statin used was Simvastatin (Sandoz Brasil´s Pharmaceutical Industry Ltd.; Cambé; PR; Brazil), and its preparation was carried out by diluting a 20-mg tablet into 20 mL of Natrosol (manufactured by compounding pharmacy Pimpinella Cosmetics; Alfenas; MG; Brazil; LOGIN: 22394928/0001-30) obtaining a final concentration of 1 mg/mL.

### Experimental periods

Ten animals from each experimental group were euthanized in a carbon dioxide chamber, which reflects an absence of odor, quick depression of the central nervous system and no residue in the animal. Euthanasia was performed at 7, 15 and 30 days post local treatments. Samples of gingival biopsies from the region with ligation were processed for biochemical analysis; the jaws were removed and sectioned in halves to be later submitted to both radiographic and laboratory processing for histomorphometric analysis.

### Cap preparation for conservation of gingival tissue samples

In order to store the samples until the time of analysis, a Tris-HCl buffer was prepared in the biochemistry laboratory of UNIFAL-MG, weighing up 3.02 grams of Tris (Hydroximethyl aminomethane – Sigma-Aldrich Corporate Offices; St Louis; Missouri; USA), 5.85 grams of sodium chloride (Vetec Fine Chemicals Ltd.; Rio de Janeiro; RJ; Brazil) and 0.555 g of calcium chloride (ACS Proquímicos - Trades of Chemicals; Santa Maria; RS; Brazil), in an analytical scale (KERN & Sohn GmbH; KERN GS 410-3; Barlingen-Frommern; Germany).

The 500-mL beaker was then filled with 250 mL of Milli-Q water (Milipore- direct Q3 UV), and it was taken to the magnetic stirrer (Fisatom - 115 V) with magnetic bar, pH meter (Digimed - DM22) and the temperature gauge (Digimed - DM 22) within, adding hydrochloric acid to the plastic Pasteur pipette to reach pH 7.5 at 25°C. The solution was added to the volumetric flask Mili-Q water until the meniscus was reached.

It was necessary to include zinc to the Tris-HCl buffer in order to carry out the sample analysis. Hence, 8.07x10^-4^ zinc sulfate (Merck) were weighed up in the analytical scale and added to a solution of 100 mL of Tris-HCl, which had been set aside in another beaker and then stirred with a magnetic stirrer using the magnetic stir bar. With the aid of a micropipette, 0.05 microliters of polyethylene glycol (Sigma Aldrich) was collected and slowly added to the buffer stirring so as not to form bubbles^22^.

### Determination of total protein concentration

Protein concentrations were determined in all the samples of gingival homogenates by the method of Bradford^4^ (1976) using bovine serum albumin (BSA) as a standard calibration curve.

The method is based on the addition of ethanol, phosphoric acid and a dye called Coomassie Brilliant Blue G-250 to a solution containing the proteins. The pH of the reaction, the interaction between the high molecular weight protein and the dye cause the displacement of the colorant to balance the anionic form (red) to the cationic form (blue), which is strongly absorbed at 595 nm^22^.

The technique consisted of using 10 uL of the solution, and the test was performed on an ELISA plate, also known as a plate with 96 cavities or wells, 36 of which were filled with samples and 11 wells were filled with reagents for calibration, called curves^11^. Readers of the ELISA plate, or micro plate readers, do spectrophotometry. They emit light at a specific wavelength and measure the amount of light absorbed and reflected by an object, where the proteins lie in the samples. The dye has a wavelength when exposed to light. The amount of reflection, absorption and the identified color, measures the amount of protein.

A spectrophotometer - using a computer program - obtained the calibration curve to the data provided, the curve equation and the correlation coefficient.

### Analysis of MMP-8 in gingival tissue

The gingival tissue collected by surgical excision using a disposable no. 15 carbon steel scalpel blade (Embramac Braz. Com. of Surgical Mat. import and Export Trade Industry Ltd.; Campinas; SP; Brazil) on the lower left molar region was stored in vials containing 2 mL of Tris-HCl buffer. This tissue was homogenized using an Ultra 80 ultrasound device (Ultra Stirrer). Then, 360 µL collected from the homogenate, 360 µL of Tris-HCl buffer solution (pH 7.5) at 4°C with 50-mM zinc sulfate (Merck KgaA; Darmstadt; Hessen; Germany) and 0.05% polyethylene glycol (Sigma-Aldrich Corporate Offices; St Louis; Missouri; USA) were added to the quartz cuvette. After that, the solution was taken to the same fluorometer (Varian Medical Systems; Mulgrave; Victoria; Australia) and 80 µL of 200-µM MMP-8 Substrate was added (Sigma-Aldrich Corporate Offices; St Louis; Missouri; USA)^22^.

The settings used in the fluorometer (Varian Medical Systems; Mulgrave; Victoria; Australia) for the analysis were 280 nm excitation and 360 nm emission. The enzyme kinetics were evaluated based on the maximum speed for the period of 0-45 minutes. Determination of MMP-8 was carried out fivefold and an average of these values was obtained for all subsequent calculations.

### Radiographic making and digital analysis

After euthanasia of the animals, the jaws were removed and fixed in 10% formalin solution for 48 hours. Then the right and left sides of the pieces were divided and the left side was submitted to an X-ray procedure.

On a table, the left hemimandibles (HMs) were positioned with the buccal surfaces facing the radiographic film (Kodak Dental Intraoral E-Speed Film; Osasco; SP; Brazil). The standardization of radiographs was obtained as follows:

Using an X-ray machine - Pampas - E (General Electric Company; Milwaukee; Wisconsin; USA), with an electric system of 65 kVp and 10 mA;

Central beam of X-rays focusing perpendicular to the plane of the film-object, at a 90° angle to the surface of the optical plate;

Focal length of 30 cm;

Exposure time of 0.8 seconds;

Radiographs were developed using Kodak developer and fixer solutions, and the time-climate developing method was used.

The radiographs were scanned and the images were analyzed with the Imagelab program (softium Computer Systems; São Paulo; SP; Brazil), using the tool for distance and angle measurement. With this feature, the distance from the cementoenamel junction to the bone crest was measured at the mesial surface of the left first molars by tracing a line, and these measurements were recorded in millimeters (mm). The mouse was positioned in the region corresponding to the cementoenamel junction. After that, the left button was triggered and dragged down to the level of the alveolar crest so that the program would automatically measure the distance.

### Laboratory processing and histometric analysis

The specimens were demineralized with 50% formic acid and 20% sodium citrate solutions and, after this step, embedded in paraffin. The cuts were performed in a semi-serial form in the mesiodistal direction. They were 4-mm thick and were stained with hematoxylin and eosin (H&E).

In order to analyze the interradicular bone level, a magnitude of up to 12X was used. The area of bone loss (BL) in the furcation region expressed in mm^2^ was histometrically determined using an image analysis system (Imagelab 2000; Software Diracon Bio Informatica Ltd.; Vargem Grande do Sul, SP, Brazil). After deletion, the first and last sections of the furcation in which the region was evident, as well as five equidistant sections of each tooth, were selected for histometric analysis^10^. The BL was assessed by measuring the area of extent between the bone crest and the surface of the furcation ceiling cement.

The selection of histologic sections was performed by a trained investigator who was blinded to the treatment performed, who also conducted the histometric analysis. The BL of each specimen was evaluated three times by the same examiner and on different days^9^. The three measurements obtained were statistically analyzed for the repeatability coefficient obtaining a reliability of 95%^28^. The mean values were statistically checked and compared.

### Statistical analysis

With a sample size of 10 (P<0.05) the power of the study was 90%. Statistical analysis of biochemical, radiographic and of the histomorphometric data obtained was performed with the BioEstat 3.0 software (Windows 1995 Bioestat Sonopress. Brazilian Industry; Manaus; AM; Brazil). The hypothesis of no statistically significant difference in the data between the different groups and periods in the teeth with induced periodontitis was tested. After examination of the normal values of the Shapiro-Wilk test, the intra and inter-group analyses were performed by analysis of variance (ANOVA) to two criteria (group and period) with the Bonferroni complementation (p<0.01).

## Results

### Analysis of MMP-8 in gingival tissue

**Figure 1:**
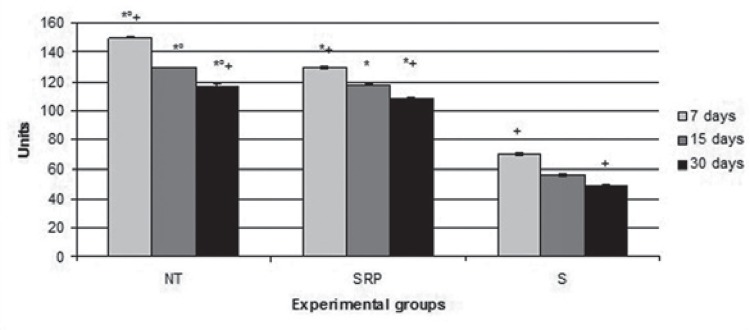
Means and standard deviations (M±SD) expression in MMP-8 units in the gingival tissue of the first lower left molar, according to each group and period

S group (70.09±0.90 un; 56.07±1.32 un; 48.59±0.30 un) had a significantly lower expression of MMP-8 compared to the NT group (150.04±0.54 un; 129.54±0.69 un; 116.76±1.01 un) and SRP group (130.03±1.33 un; 117.37±0.40 un; 108.33±1.23 un) in all experimental periods (p<0.01). The SRP group had a significantly lower expression of MMP-8 compared to the NT group in all experimental periods (p<0.01). The animals of all groups showed a significantly lower expression of MMP-8 at 30 days when compared to expressions at 7 days (p <0.01) (Figure 1).

*Difference with S group, the same period (ANOVA and Bonferroni, p<0.01).

°Difference with SRP group, the same period (ANOVA and Bonferroni, p<0.01).

+Difference between periods, the same groups (ANOVA and Bonferroni, p<0.01).

### Radiographic analysis

**Figure 2:**
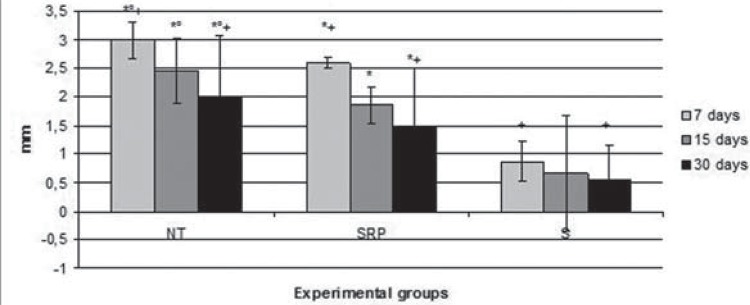
Means and standard deviations (M±SD) of the distances between the cementum-enamel union and alveolar bone crest (mm) on the mesial surface of the first lower left molar, according to each group and period

In the radiographic analysis between the groups, the S group (0.86±0.34 mm; 0.66±1.01 mm; 0.57±0.23 mm) had a significantly lower bone loss (BL) compared to the NT group (2.99±0.32 mm; 2.45±0.58 mm; 1.97±1.11 mm) and the SRP group (2.59±0.09 mm; 1.86±0.33 mm; 1.49±1.02 mm) in all experimental periods (p<0.01). The SRP group had a significantly lower BL compared to the NT group in all experimental periods (p<0.01). The animals of all groups showed a significantly lower BL at 30 days compared to 7 days (p<0.01) (Figure 2).

*Difference with S group, the same period (ANOVA and Bonferroni, p<0.01)

°Difference with SRP group, the same period (ANOVA and Bonferroni, p<0.01)

+Difference between periods, the same groups (ANOVA and Bonferroni, p<0.01)

### Histometric analysis

**Figure 3:**
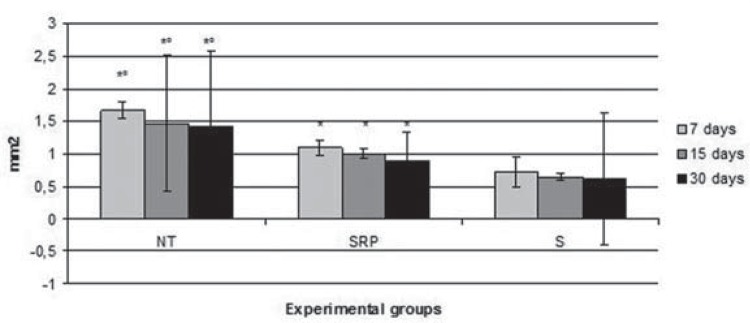
Means and standard deviations (M±SD) of hypsometric data PO (mm2) in the furcation region of the left first molars, according to each group and period

**Figure 4:**
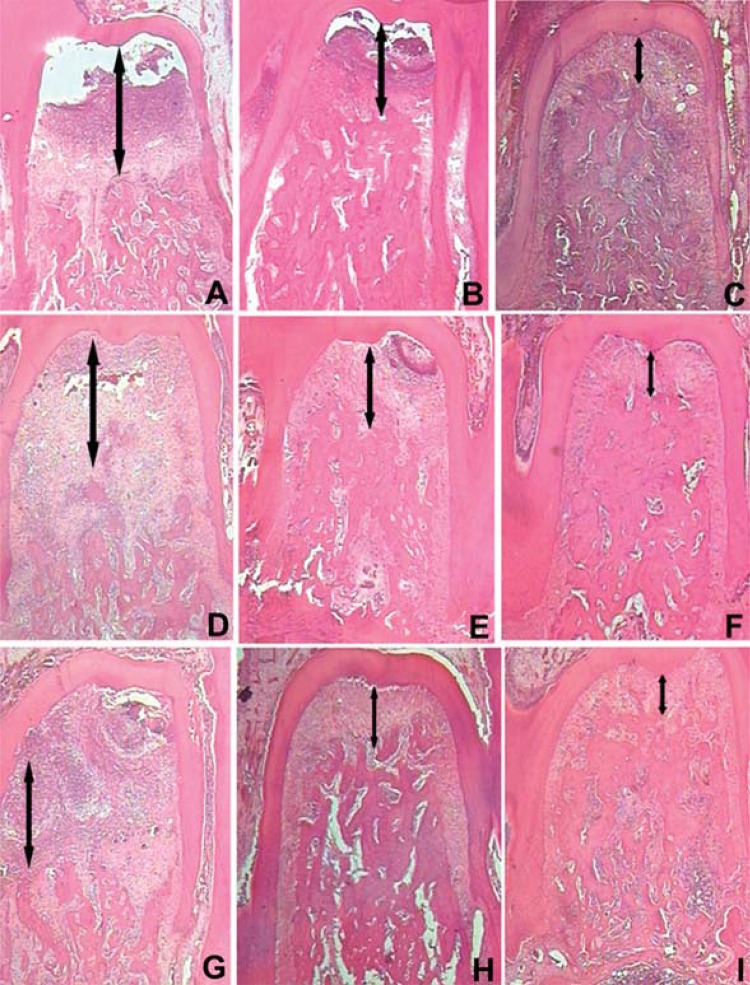
Photomicrographs illustrating the areas of BL in the furcation region of the left first mandibular molars with induced periodontal disease. Original magnification 12X. HE. A – NT group, 7 days; B - SRP group, 7 days; C- S group, 7 days; D – NT group, 15 days; E - SRP group, 15 days; F- S group, 15 days; G – NT group, 30 days; H - SRP group, 30 days; I- S group, 30 days

In histometric analysis between groups, the S group (0.71±0.23 mm^2^; 0.64±0.05 mm^2^; 0.61±1.02 mm^2^) had a significantly lower bone loss (BL) compared to the NT group (1.67±0.13 mm^2^; 1.47±1.05 mm^2^; 1.41±1.17 mm^2^) and the SRP group (1.09±0.10 mm^2^; 1.00±0.08 mm^2^; 0.89±0.45 mm^2^) in all experimental periods (p<0.01). The SRP group had a significantly lower BL compared to the NT group in all experimental periods (p<0.01) (Figure 3 and Figure 4).

*Difference with S group, the same period (ANOVA and Bonferroni, p<0.01)

°Difference with SRP group, the same period (ANOVA and Bonferroni, p<0.01)

## Discussion

The mechanical removal of dental plaque by SRP is the base of periodontal treatment, but may be flawed in some dental sites^2^. Thus, the aim of this study was to evaluate the local effects of statins as an adjuvant treatment to SRP in periodontal disease induced in rats. The results of this study showed a slight asymmetry, probably related to variations between the animals and the processing used to obtain the biochemical, radiological and histometric data. However, when performing statistical tests it was observed that it had been a normal distribution of data (parametric).

The choice of rats as an experimental animal model was based on other studies^14^. According to the author, the rat is favorable as an experimental model for the development and study of periodontal disease due to the similarity of their periodontium to that of humans, where the only difference is the presence of keratinization of the sulcular epithelium. The model of periodontal disease induction used in this study was the same proposed in a former study carried out in 1975^13^, which used the placement of a cotton thread around rat molars. The author pointed out that the bandage favors bacterial accumulation, thus developing periodontal disease. In the present study, this model was also efficient in the induction of experimental periodontal disease because of the ligature induced plaque formation and a local inflammatory response. Periodontal disease is characterized by clinical signs of gingival inflammation, such as swelling, redness and loss of adhesion of gingival tissue to the tooth.

Spontaneous healing after ligature removal by lack of mechanical treatment (SRP) in PD noticeably promoted intense alveolar bone resorption in the furcation area resulting from the maintenance of the primary etiologic agents of periodontitis, as shown by the histometric analysis at all time points. Periodontitis is characterized by epithelial migration and by the breakdown of connective tissue and alveolar bone^30^. Thus, the lack of mechanical treatment contributes to the progression and evolution of PD in the present study. The data presented here clearly demonstrate PD remission after SRP treatment, thus corroborating the consensus in the literature that SRP treatment is effective in periodontitis remission^9^ compared with the free progression of periodontitis, wherein the removal of the aggressor is not performed by mechanical treatment. Although the SRPs exhibit satisfactory results in the present study, excessive penetration of instruments in the periodontal pocket bottom can lead to an insertion loss in teeth with little probing depth^17^. However the benefits of penetration of periodontal instruments in the bottom of the bag, directed for the removal of bacterial deposits present on the root surface, are infinitely larger than the damage caused by the trauma produced^2^.

The use of adjuvants for the periodontal treatment was aimed to decrease or eliminate microorganisms that contribute to the onset or progression of periodontitis. Pharmacologic agents for systemic^29^ and topical^13^ use are applied for this purpose. Studies^19^ ^,^ ^20^ have addressed the use of substances that affect the activity of osteoclasts because these cells cause alveolar bone breakdown in periodontitis^20^. Accordingly, statins, which are drugs that inhibit the action of osteoclasts, are considered potent agents in the control of bone resorption^21^ ^,^ ^23^. In the present study, periodontal pockets were irrigated with 1 mL Simvastatin gel after SRP. Systemic effects of the accidental ingestion of Simvastatin by animals during local treatments could cause lowering cholesterol levels, flatulence, diarrhea, constipation, nausea, and the emergence of other unpleasant reactions^5^. But these changes were not observed in the animals of this study because during the treatments the oropharyngeal region of each pet was protected with a sterile gauze thereby preventing the intake of any gels and preventing the systemic action of simvastatin.

Matrix metalloproteinases (MMPs) play an important role in tissue remodeling during development, homeostasis and wound healing^26^. An imbalance between MMPs and their activated derivatives of the host endogenous inhibitors, the inhibitors of matrix metalloproteinase (TIMP), leads to the pathological breakdown of the extracellular matrix in periodontal disease^26^. Among other activities, MMPs degrade collagen fibers inserted into the tooth root, allowing apical migration and lateral extension of the pocket epithelium. The clinical sequelae of the pathologic increase in collagen destruction are the insertion loss, bone loss and formation of periodontal pockets^1^. MMP-8 has aroused interest for it has been found in various inflammatory diseases, such as periodontitis, bronchitis, asthma and arthritis. Recently it has been shown to exert an unexpected defensive anti-inflammatory activity, which provides protection against experimental skin cancer scattering and pulmonary inflammatory disease, probably rendering anti-inflammatory cytokines and chemokines as well as regulating the apoptosis of inflammatory cells and the immune response^12^. Along with MMP-9 and neutrophil-derived MMP-13 derived from bone or skin cells, MMP-8 stands out among MMPs predominantly present in inflamed gingival tissue, FGC, saliva and crevicular fluid in peri-implants. The level and degree of activation of these enzymes seems to increase with the increased activity and severity of periodontal disease, and then decreases after the treatment with scaling and root planing (SRP)^12^.

A recent study showed that MMP-8 levels in patients that were both smokers and non-smokers with chronic periodontitis have significantly reduced after periodontal therapy SRP^1^. This was demonstrated in the present study, considering that in MMP-8 expression intra-groups, the animals of all groups showed a significantly higher expression at 7 days compared to 30 days after local treatments.

It is also possible to observe that animals in the S group had expressions of MMP-8 that were significantly decreased compared to the SRP group in all experimental periods, probably due to the anti-inflammatory action of Simvastatin. A study conducted in the current year^16^ strengthens this hypothesis, since it demonstrated that topical application of Simvastatin was able to reduce the inflammatory process in experimental periodontitis in rats. In 2003, Luan and collaborators^18^ found that statins decrease the production of many pro-inflammatory cytokines, and it has been described that they favor the decreased secretion of other matrix metalloproteinases *in vitro*. Thus, they could reduce the inflammatory response, providing protection against the destruction of periodontal tissue.

Statin drugs are inhibitors of the enzyme 3-hydroxy-3-methyl-glutaryl-coenzyme A (HMG-CoA) reductase, leading to the production management of cholesterol^24^ ^,^ ^25^ ^,^ ^27^. Among the various statins, Simvastatin, which has been widely used in clinical practice to control cholesterol, has proven to be pharmacologically safe. In addition to its hypolipidemic function, this statin is notable for other side effects, including anti-inflammatory^27^, immunomodulatory and antioxidant properties, as well as the promotion of angiogenesis and increased osteoblast differentiation, inducing bone formation^6^. These properties provide great potential for statins to modify the course of chronic inflammatory diseases^3^, among which chronic periodontitis may be included.

Statins have different effects on bone, such as increasing formation. Hence, the choice of Simvastatin in the present study was considered since the effects shown are more intense^3^, as would also be the effect with Atorvastatin and Cerivastatin. Conversely, Lovastatin and Pravastatin exhibit little effect. In addition, Simvastatin stands out when acting at major events during an exacerbated inflammatory response, as mentioned before.

In the radiographic and histometric analyses between groups, the S group had a significantly lower BL compared to the NT and SRP groups at 7, 15 and 30 days, respectively. Published studies have also shown beneficial effects of statins in preventing bone loss in experimental periodontitis in rats^16^ ^,^ ^19^. One of the factors that could explain these results is that statins inhibit the transformation of pre-osteoclasts in osteoclasts through bone marrow cells and osteoblasts via indirect action. Osteoblasts recruit and activate osteoclasts by RANKL interaction on the surface, with the RANK receptor in the hematopoietic precursor cells of osteoclasts. This osteoclast activation is controlled by osteoblasts by means of the OPG secretion of a soluble receptor that competes with RANK for RANKL to inhibit the recruitment of osteoclasts, maintaining a balance between them^25^.

The action of statins derives also from the inhibition of osteoblast apoptosis by the action of transforming growth factor beta (TGF-β). TGF-β has a key role in bone formation, with Smad proteins (“mothers against decapentaplegic”) belonging to this signaling pathway^29^ ^,^ ^30^. There is evidence of osteoblast apoptosis inhibition regulated by Pitavastatin, Mevastatin and Simvastatin, due to the higher expression of Smad 3 (“mothers against 3 homolog decapentaplegic”), which functions as a signal transducer and a modulator of transcription^21^ ^,^ ^23^ ^,^ ^30^. The active Smad 3 is crucial to maintain bone formation, while its suppression leads to osteoblast apoptosis^23^ ^,^ ^30^.

Considering the fact that this study has been carried out on animals, which showed a slight asymmetry of data (although the data is parametric) it would be unwise to extrapolate the results to the human species; thus, further studies of the literature are needed. Therefore, within the limits of this study, it is possible to conclude that a locally applied statin was effective as an adjuvant treatment to the SRP in induced periodontal disease in rats.
